# Making sense of PROM outcomes: a mixed method study to optimize graphical visualization formats for children

**DOI:** 10.1007/s11136-026-04172-5

**Published:** 2026-02-12

**Authors:** Selina Limmen, Maud M. van Muilekom, Dorinde L. Korteling, Lorynn Teela, Maarten Boers, Caroline B. Terwee, Hedy A. van Oers, Lotte Haverman, Michiel A. J. Luijten

**Affiliations:** 1 Child and Adolescent Psychiatry & Psychosocial Care, Amsterdam UMC location University of Amsterdam, Emma Children’s Hospital, Amsterdam, The Netherlands; 2 Amsterdam Reproduction and Development, Child development, Amsterdam, The Netherlands; 3 Amsterdam Public Health, Mental health, Amsterdam, The Netherlands; 4 Amsterdam Public Health, Personalized Medicine, Amsterdam, The Netherlands; 5 Amsterdam Public Health, Methodology, Amsterdam, The Netherlands; 6 Amsterdam Public Health, Digital health, Amsterdam, The Netherlands; 7 Department of Epidemiology and Data Science, Amsterdam UMC, Vrije Universiteit, Amsterdam, The Netherlands; 8 Amsterdam Public Health, Quality of Care, Amsterdam, The Netherlands

**Keywords:** Patient reported outcome measures, Visualizations, Graphics, Pediatric, Interpretation, Comprehensibility

## Abstract

**Objective:**

Graphs are often used to increase patient understanding of Patient Reported Outcome Measure (PROM) scores. We aimed to investigate which graph visualization options are best interpreted by children.

**Methods:**

We conducted a quantitative study to assess children’s interpretation accuracy and perceived clarity of different PROM visualizations graphs through a test, and a qualitative study using ‘think aloud’ interviews about the same visualizations to explore how different visual elements were used for interpretation. Participants included (1) children from the Dutch general population (quantitative) and (2) children (8-18y) treated at Emma Children’s Hospital (quantitative and qualitative).We created sample graphs (e.g. bar, line) based on literature, varied graphical elements (e.g. addition of numerical information yes/no), and presented them in randomized order. Logistic and linear mixed models analyzed interpretation accuracy and clarity ratings. We analyzed interviews with a deductive approach.

**Results:**

We included 558 children in the quantitative study (1: 546, 2: 12), and 14 in the interviews. Foundational elements like clear labeling were found beneficial. ‘Heatmap’ and ‘color’ as indicators of concerning scores had higher interpretation accuracy and clarity, and SD-lines lower. Bar graphs had slightly higher accuracy. Radar graphs scored significantly worse on interpretation accuracy and clarity compared to all other graphs. Interviews revealed a preference for single domain graphs with color-coding indicating concerning scores.

**Conclusion:**

We recommend using bar graphs incorporating heatmaps or color as concerning score indicators in clinical encounters with children. Application of our results will likely facilitate patient engagement in consultations and potentially improve patient-centered care.

**Supplementary Information:**

The online version contains supplementary material available at 10.1007/s11136-026-04172-5.

## Introduction

Patient-reported outcome measures (PROMs) are standardized questionnaires that assess patients’ self-reported physical, mental, and social health [1]. They provide valuable insights into issues that matter most to patients and are increasingly integrated into clinical practice to monitor patients’ symptoms and functioning [2–4], tailor interventions [5, 6], enhance communication between the patient and healthcare provider, and improve quality of life [7]. 

To facilitate remote PROM completion and use in daily care, online PROM portals have been developed [8]. PROM use in pediatric care demonstrated increased discussion of psychosocial topics and patient satisfaction [8]. However, implementation is more challenging than in adult care due to the wide range of developmental stages. Pediatric PROM portals, such as the KLIK PROM portal (www.hetklikt.nu) [8, 9], provide age-appropriate PROMs and proxy versions for caregivers when children are too young or unable to self-report. To ensure privacy, pediatric PROM portals often provide separate accounts for children and parents. From about eight years of age, most children can reliably complete PROMs themselves, which is preferred over proxy reporting as it better reflects the child’s own perspective on health and quality of life [10]. 

PROM portals return results to healthcare providers via literal responses and/or graphical visualizations. To fully leverage the benefits of PROM use and enhance patient engagement, clinicians are recommended to review PROM results together with patients during consultations. For patients to engage in these discussions, PROM results need to be presented in a clear and interpretable way [11–14]. However, designing appropriate PROM visualizations is challenging due to the large variety of PROMs [15], lack of standardization in scoring and graphical representation [16], and limited knowledge on how children interpret graphical feedback. In the early development of KLIK for example, children reacted to upward or downward trends in line graphs, expressing sighs of relief or disappointment [9], resulting in discontinuation of further development of child-directed PROM visualizations. Research mainly focused on PROM visualizations for clinicians, with a few studies in adult patients [17, 18]. 

Interpretability of a graph depends on both basic elements (e.g., clear axis labels and titles) and the choice of graph type [19]. In adult patients, line and bar graphs were among the most interpretable graphical formats [12, 13, 16, 18–20]. Key features that enhance interpretation include directionality (e.g. higher scores indicating better outcomes), and features to indicate scores of concern, such as threshold lines [13, 16, 19]. In contrast, studies in children have so far been limited to preferences for a small set of line graph options, without addressing actual interpretation accuracy [21, 22]. This is an important gap, as preference does not necessarily translate into correct understanding. Children in the Netherlands are introduced to basic graphs such as simple bar graphs from age six, and more complex graphs including line graphs from age eight [23]. While studies suggest that children can interpret such graphs when given sufficient time and support [24], challenges remain, particularly with line graphs [25]. The ability to interpret graphs is influenced by the visual features of the graphs, as well as the child’s prior knowledge and expectation about the data [26]. 

There is a clear lack of knowledge regarding how to effectively present PROM results to children. Therefore, this study aims to assess the interpretation accuracy and clarity of different graphical formats of PROM visualizations for children. The results are intended to inform recommendations for the optimal design of PROM visualizations for pediatric use.

## Methods

### Study design

This mixed methods study has a convergent parallel design, combining quantitative and qualitative parts [27]. The quantitative part employed a test containing assignments to investigate interpretation accuracy and perceived clarity of sample PROM graphs in a large group of children. The qualitative part comprised interviews about the same test, using the *think aloud* method [28] to explore how different visual elements were used to interpret graphs. *Think aloud* is a commonly used method to investigate participant interaction with visualized information [29], where children are encouraged to verbalize their thought process. The Medical Ethics Review Board of the Amsterdam UMC, location AMC waived the requirement for approval under Dutch Law on research with humans (2024.0017).

### Participants and procedure

We included two participant groups in this study: (1) children aged 8–18 years from the Dutch general population recruited through marketing agency Panel Inzicht, and (2) pediatric patients aged 8–18 years, treated at the Emma Children’s Hospital and part of the KLIK panel (KLIK PROM portal users who consent to be contacted for research (*n* = 607)). The age threshold was set at 8 years because children in the Netherlands are introduced to various graph types in school at this age [23], and are deemed capable of self-reporting PROMs reliably [30]. Non-Dutch speakers and children with intellectual disability were excluded.

For the quantitative study we aimed to include at least 500 children from the general population, and 50 pediatric patients. General population participants were recruited through Panel Inzicht, which emailed parents of its panel members with a link to the test. Pediatric patients were recruited by emailing parents of children included in the KLIK panel. As we wanted to use this panel for both quantitative tests and the think aloud sessions, we approached only half of the panel for the quantitative test (*n* = 304). The quantitative part of this study ran between September and December 2024. The test was conducted through an online research website of the KLIK PROM portal (www.prom-datavisualisatie.nl). All participants provided written consent for participation (children < 16 years alongside caregiver consent). After the quantitative study, children from the general population received a small participation fee via Panel Inzicht, and pediatric patients received a €5 gift voucher by email.

For the qualitative interviews, we aimed to include 10–15 pediatric patients, with the final number guided by whether the data were sufficient to address the research question [31]. The remaining half of KLIK panel members (*n* = 303) were invited by email to participate in the qualitative interviews. Interviews including one participant and a researcher experienced in qualitative research [ML, MM, SL or LS] were held live at the Amsterdam UMC in November 2024 and lasted 45 min. Children were asked whether they felt comfortable completing the interview without parental presence. If a parent remained present, they were seated out of view of the screen and instructed not to assist. Participants completed the test through the online research website while verbalizing all their thoughts aloud. The researcher posed in-depth questions when necessary. All sessions were audio recorded. Interview participants received a €15 gift voucher.

### Measures

#### Sociodemographic information

Parents and children completed a sociodemographic questionnaire about the child, with questions about age, gender, country of birth, and educational level.

#### Test

We based PROM visualizations on the domains and scoring of the Patient-Reported Outcome Measurement Information System (PROMIS^®^), where the mean equals 50 and cut-off values for concerning scores are 10/20 points deviation from the mean [32]. 

**Fig. 1:**
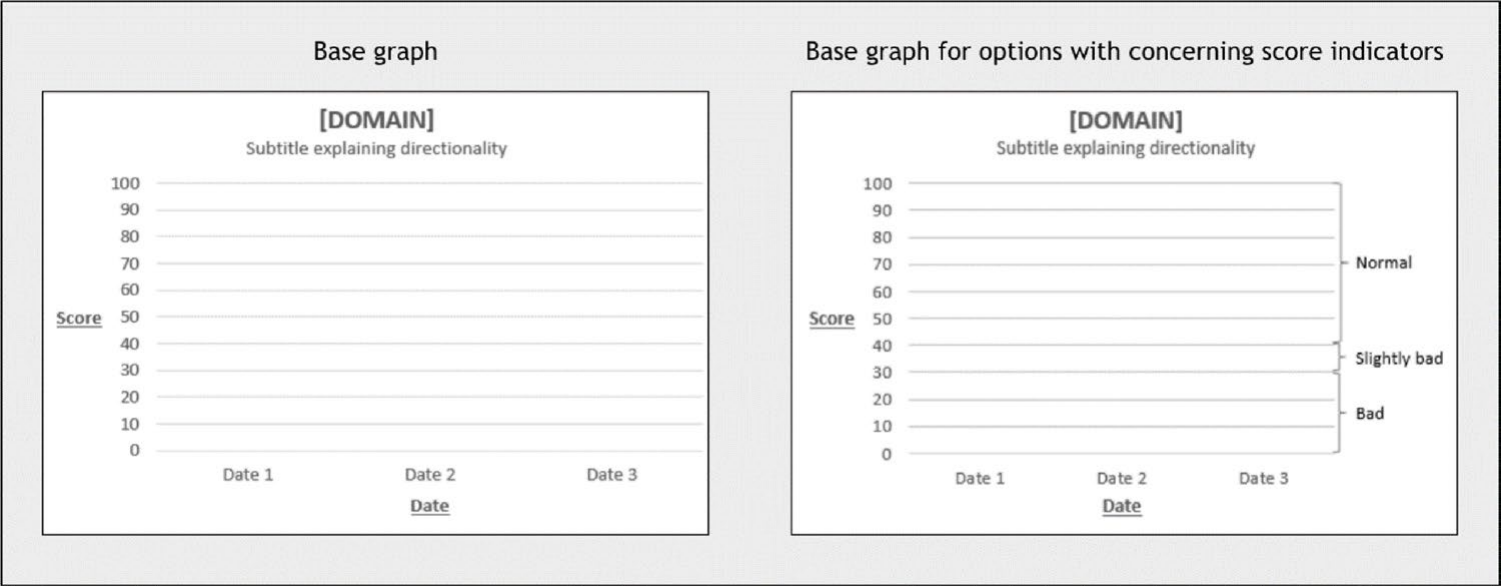
Base graph based on common standards of data visualization

Prior to developing the test, we employed expertise within the author team (MB) to develop a base graph in accordance to common standards of data visualization (Fig. 1) [19]. This included the use of light grid lines, proper scaling and labeling of the axes and the inclusion of a proper title and subtitle explaining content and directionality (“Line going up means improvement”). For graphs including concerning score indicators (i.e. cut-offs; indicators for mild/severe problems), bracketed labels on the right y-axis indicated “normal,” “slightly bad,” and “bad” score ranges. All graphs were designed in Microsoft Excel (version 2408).

To minimize learning effect and participant burden, we created four distinct test versions (sets) (Table 1). All sets contained two parts; Part A focused on single-domain graph samples, with manipulation of graphical features based on research among adults. Part B included graphs displaying multiple domains in one graph that are commonly used in clinical practice.

**Table 1:** Test sets

	Set 1		Set 2		Set 3		Set 4	
*Part A: single domain*								
Numerical information	No		Yes		No		Yes	
Directionality	Higher is better*				Higher is more†			
Graphs	Bar	Line	Bar	Line	Bar	Line	Bar	Line
Concerning score indicators								
heatmap		+ (pain)	+	+	+	+	+ (pain)	+ (pain)
colored bars/symbols		+ (pain)	+	+	+	+	+ (pain)	+ (pain)
SD-lines	+	+		+ (pain)	+ (pain)	+ (pain)	+	+
none	+	+		+ (pain)	+ (pain)	+ (pain)	+	+
*Part B: multiple domains*								
Numerical information	No		Yes		No		Yes	
Directionality	Higher is better							
Graphs	Bars; Lines; Balloons; Radar		Bars; Lines; Balloons; Radar		Bars; Lines; Balloons; Radar		Bars; Lines; Balloons; Radar	

**Fig. 2:**
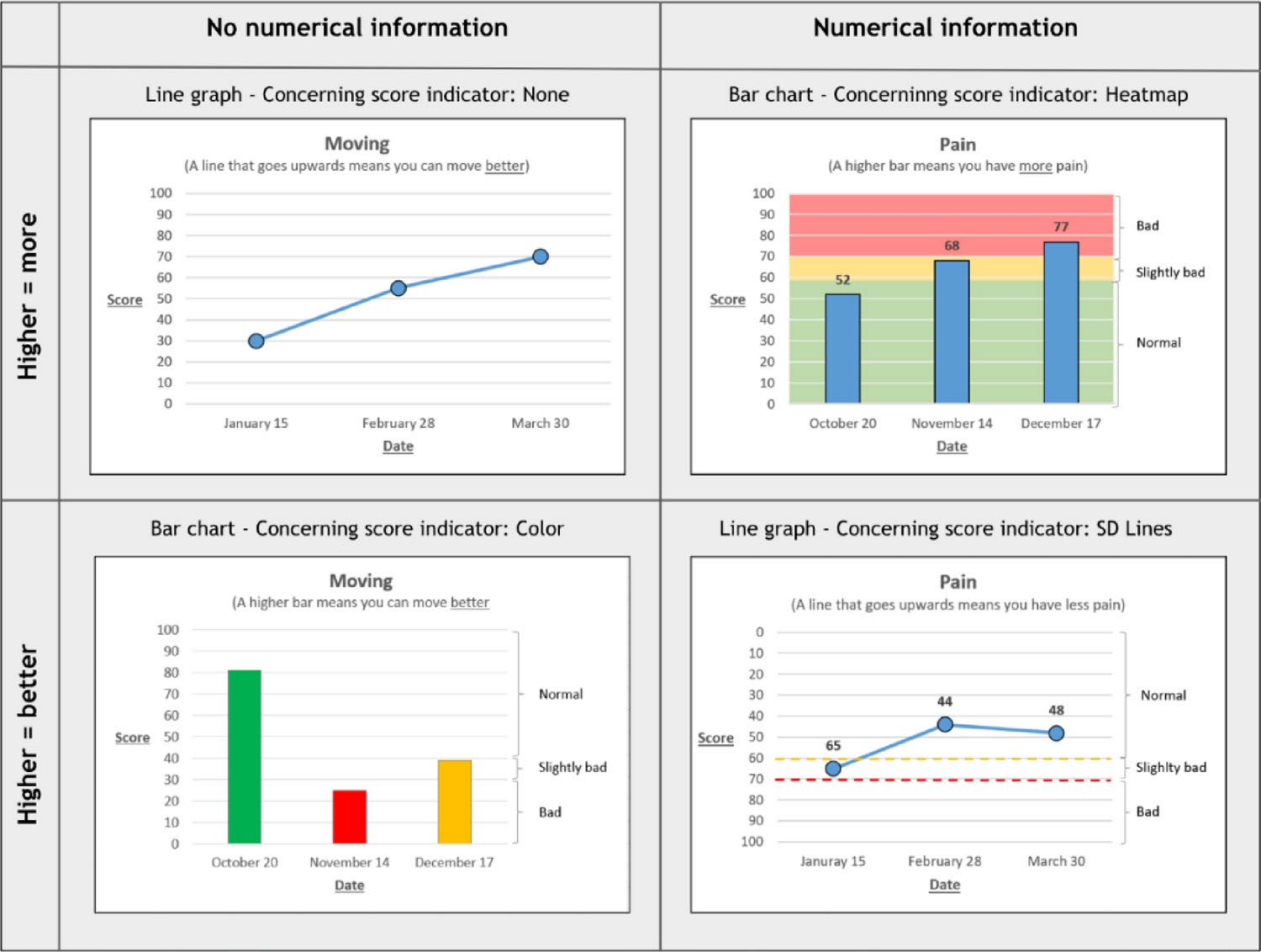
A random selection of graph examples from Part A of this study, demonstrating differences in manipulated visual elements

Part A examined line and bar graphs incorporating interpretative features previously shown to be most interpretable for adults [12, 13, 16, 18–20]. Features varying across sets were numerical information (presentation of numerical PROM domain scores within the graph; yes/no), directionality of the y-axis (higher = a better outcome or: higher = more of the domain), and presence and form of concerning score indicators (heatmap, colored bars/line graph dots, SD-lines, none). Graphs presented domain scores of PROMIS Pain or Mobility. We used simple words to describe the PROMIS domains in the graphs to minimize effects of (health) literacy; mobility was translated to ‘moving’. Each graph showed three datapoints (dates of PROM completion). Domain scores of these datapoints were equal within features across sets. Participant viewed 6–8 graphs for Part A. As bar graphs cannot accommodate reversed y-axis directionality (i.e. if 100 is at the bottom of the y-axis, the bar would come from above), symptom domains where higher scores indicate worse outcomes could only be presented in the “higher is more” format. Examples of Part A graphs are presented in Fig. 2.

**Fig. 3:**
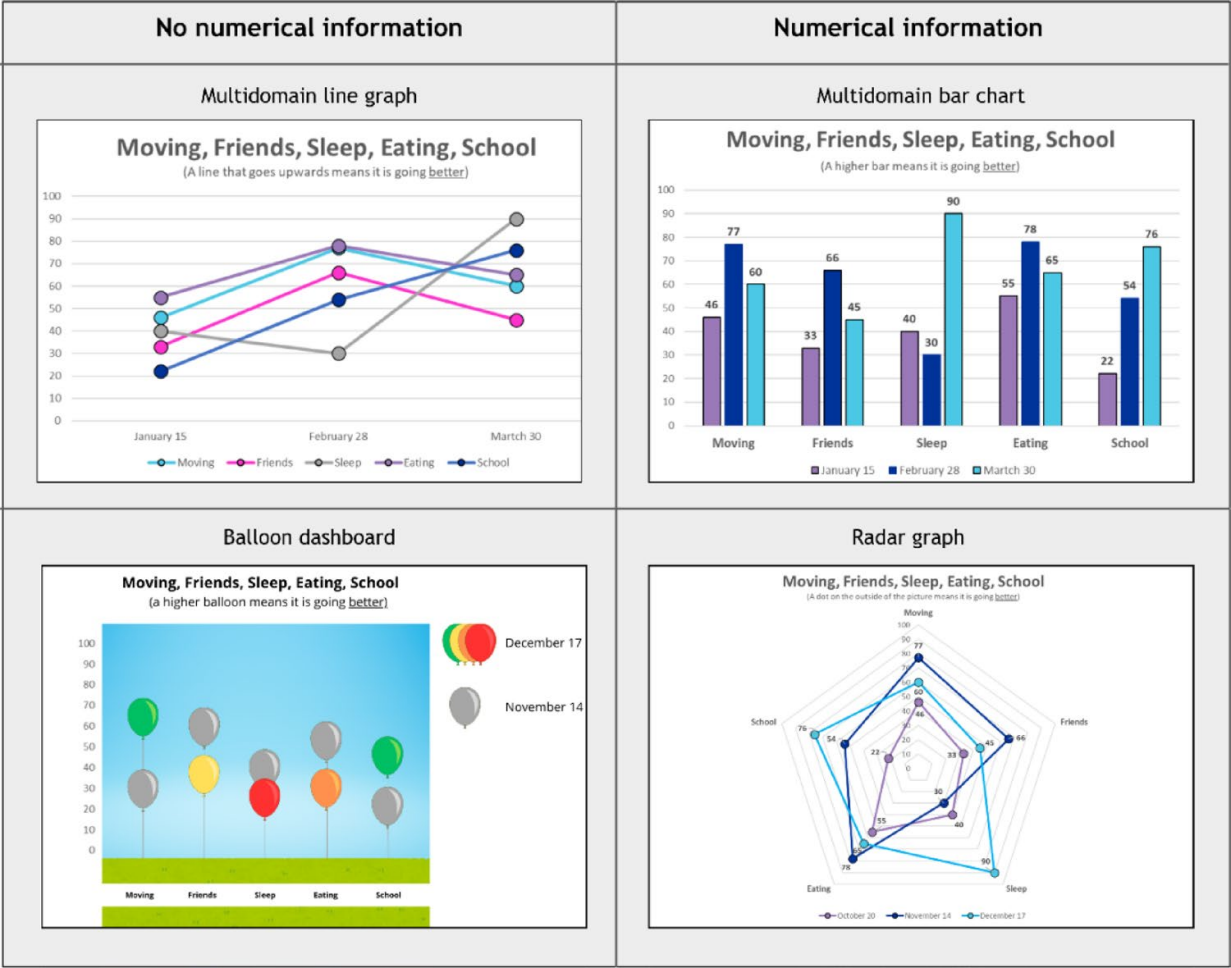
A random selection of graphs examples for Part B of this study

Part B examined a sample of graph types specifically developed to present data across multiple domains: multidomain bar and line graphs, balloon dashboard [33], and radar chart [34]. Graphs presented PROMIS domains Mobility (‘moving’), Peer relationships (‘friends’), Nutrition (‘eating’), Sleep disturbance (‘sleeping’) and School functioning (‘school’). Only numerical information was included as optional feature due to the diverse nature of the graphs. All sets included all multidomain graphs. Examples of Part B graphs are presented in Fig. 3.

**Fig. 4:**
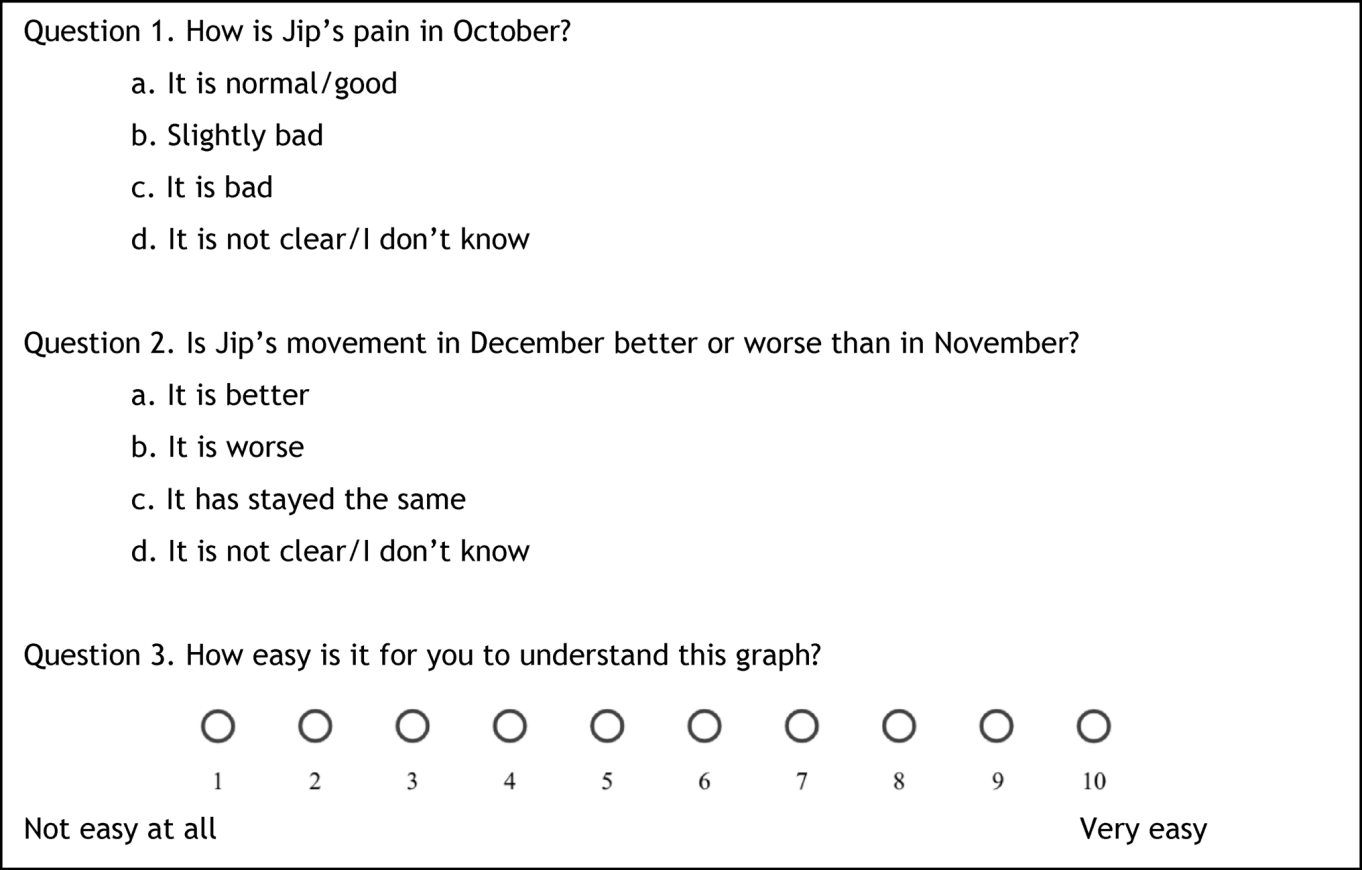
Test question examples for the selected domain “Pain”

The children advisory board of the Emma Children’s Hospital contributed to formulating the test introduction. In part A, participants viewed each graph of one set and answered three questions (Fig. 4). The first two assessed interpretation accuracy: question one asked how the person of whom the data was depicted in the graph was doing in a specific month compared to normative data, and question two asked if the person improved or deteriorated between the two mentioned months. Question three focused on clarity of the graph.

In Part B, participants answered question two (changes over time), and question three (clarity). Graphs were shown in random order within part A and B separately. After part B, all graphs from both part A and B were displayed and the children selected their preferred option.

Participants were not shown whether their responses were correct. 

### Analysis

We provided descriptives of the sociodemographic characteristics for all separate samples and the total sample.

#### Quantitative study

Test responses were ‘cleaned’ prior to analyses to preserve quality by removing participants using the same answer option throughout the entire test (flatliners), as this pattern suggests limited participant engagement and compromises the validity of the data [35]. We made an exception for participants using only response option d: *‘I don’t know’*. Participants’ answers to multiple choice questions were categorized as correct, incorrect, or ‘*I don’t know’* (response option d). Chi-square tests of independence assessed whether the proportion of correct responses differed significantly between pediatric patients and the general population. We used IBM SPSS statistics 28 in all analyses.

##### Single domain graphs

We computed the percentage correct, incorrect, *‘I don’t know’* responses for each graph. For further analysis, we reclassified the response option *‘I don’t know’* as incorrect, reflecting unsuccessful interpretation of the graph. To assess the associations between the different graphical features we performed a multivariable logistic regression with generalized estimating equations with participant ID to account for within-subject effects. The dependent variable was a correct answer (0 = incorrect, 1 = correct) to a question on interpretation accuracy. Independent variables included in the model were the following graphical features that were manipulated between options: numerical information, directionality, format, and concerning score indicators. Concerning score indicators were included as nominal variable (heatmap, colors, SD-lines, none), where ‘none’ was used as reference category. We corrected for participant age by including it as covariate in the model. We performed the multivariable logistic regression for question one and question two separately, as we expected that some features may lend themselves better to longitudinal interpretation than others. We calculated odds ratios (ORs) to quantify the association between each graphical feature and the odds of giving a correct answer. We interpreted ORs between 0.5 and 2.0 as small to moderate effects, and those below 0.5 or above 2.0 as large effects, provided they were statistically significant (*p* ≤ 0.05) [36]. 

We analyzed the association of graphical features with clarity ratings of graphs (question three) using a linear mixed model with participant ID to correct for within-subject effects. We included the same independent variables and covariates in the model as for the previous analysis. If we found a significant association of concerning score indicators with clarity, we performed post-hoc comparisons with Bonferroni correction to determine which indicators differed from each other. We calculated B-coefficients for each graphical feature, and interpreted them in relation to the clarity rating (range 0–10), considering B ≥ 0.5 as a large effect. B values < 0.5 were interpreted as small to moderate effects. Effects were considered significant with a *p*-value < 0.05.

#### Multidomain graphs

We calculated the percentage of correct, incorrect *‘I don’t know’* responses for each graph type (multidomain bar and line graphs, balloon dashboard, and radar chart). For further analysis, *‘I don’t know’* was reclassified as incorrect.

Prior to assessing differences in interpretation accuracy and clarity of the different graph types, we assessed whether the inclusion of numerical information was associated with more correct responses. If this was not the case, we could combine the graph types with and without numerical information, resulting in five rather than ten graph types. We performed a one-way ANOVA with post-hoc testing to detect differences in correct responses between the different graph types. In addition, we calculated the mean and standard deviation of the clarity ratings for each graph type.

##### Single versus multidomain graphs

We conducted a chi-square test to compare correct responses between single domain bar and line graphs from part A, and multidomain bar and line graphs from part B.

##### Preferred graph option

We reported the percentage of participants who selected each format as their preferred option.

#### Interviews

Transcriptonline transcribed the interview recordings and MAJL checked the transcripts. We analyzed with a deductive approach using MaxQDA. Before analyzing, we developed a code tree based on the visual elements of the graphs, including both base graph components and manipulated features (i.e. features included in the multivariable analyses). Participant remarks were coded by assigning descriptive codes reflecting the content of the remark per visual element, which were subsequently classified as “positive”, “negative” or “missing/suggestion”. Two authors (SL & MAJL) independently coded 10% of the interviews and had consensus meetings to consolidate results. After consensus and confirmation that the deductive coding was successful, the remaining interviews were coded by MAJL. Think-aloud findings are integrated throughout the relevant sections of the results, with overarching themes presented separately.

## Results

A total of 577 participants started the quantitative study; 19 were excluded due to lack of response variability, resulting in 558 participants (including 44 partial completions). Of these, 546 participants were from the general population and 12 were pediatric patients from the KLIK panel. Pediatric patients had a significantly higher percentage of correct responses (76%) over the test compared to the general population (67%) (*p* = 0.001). Due to the small pediatric patient sample, further analyzes were conducted with the samples of the general population and pediatric patients combined. Fourteen pediatric patients from the KLIK panel participated in the interviews. Sociodemographic characteristics are shown in Table 2.

**Table 2:** Sociodemographic information

Sociodemographic information	Quantitative study participants				Interview participants		All participants (*n* = 572)	
	General population (*n* = 546)		Pediatric patients^ (*n* = 12)		Pediatric patients^ (*n* = 14)			
*Age in years*								
Mean [SD]	12.9 [2.8]		11.8 [3.2]		11.5 [2.6]		12.9 [2.8]	
Range	8–18		8–18		8–17		8–18	
Sex	*count*	*%*	*count*	*%*	*count*	*%*	*count*	*%*
Male	284	52	5	42	9	64	298	52
Female	261	48	7	58	5	36	273	48
Different / Prefer not to say	1	0	0	0	0	0	1	0
*Country of birth*								
Netherlands	533	98	12	100	14	100	559	98
Other	13	2	0	0	0	0	13	2
Current educational level*								
Primary	185	34	7	62	9	64	201	35
Secondary	301	55	5	35	4	29	310	54
Tertiary	53	10	0	0	0	0	53	9
Other	7	1	0	0	1	7	8	1

* Primary education: foundational stage of formal learning for basic skills, typically from around age five to twelve. Secondary education: advanced general education around ages 12–18. Tertiary education: higher education (universities, colleges) and vocational education

^ Pediatric patients were treated by different healthcare teams within the Emma Children’s Hospital: follow-up neonatology, follow-up intensive care, cleft care, endocrinology, kidney transplantation, rheumatology, connective tissue disorders, hematology, ulcerative colitis, sickle cell disease, and gastroenterology

### Overarching results from interviews

Basic graphical elements, such as labeling of the axes, titles, and subtitles were often used to aid interpretation. All children used the subtitle to determine directionality. Most children used the labels on the y-axis describing zones of severity to interpret the scores.

### Single domain graphs

#### Interpretation accuracy

Supplementary 1 presents the percentages of correct, incorrect, and ‘I don’t know’ responses for each graph. The associations between different graphical features and correct responses are shown in Table 3.

##### Comparison with norm population

Compared to the reference condition (‘None’), the concerning score indicators heatmap (OR = 2.89, *p*  = < 0.001) and color (OR = 1.97, *p* < 0.001) were significantly associated with higher odds of a correct response, while SD-lines were significantly associated with lower odds (OR = 0.68, *p* < 0.001). Additionally, format and directionality showed small but significant associations with interpretation accuracy, with bar graphs leading to more correct responses than line charts (OR = 1.23, *p* < 0.001), and the directionality higher is better resulting in better outcomes than higher is more (OR = 1.30, *P* = 0.005). Numerical information was not significant associated.

##### Changes over time

Analyses showed a significant association of concerning score indicators on interpretation accuracy (*p* < 0.001). Compared to the reference condition (‘None’), SD-lines were significantly associated with lower odds of a correct response (OR = 0.65, *p* < 0.001). No other variables showed significant associations.

#### Clarity

Corresponding with the above results, pairwise comparisons showed that concerning score indicators heatmap and color were associated with significantly higher clarity ratings compared to ‘None’ (heatmap (B = 0.48, *p* < 0.001), color (B = 0.33, *p* < 0.001)). Bar graphs were associated with significantly higher clarity ratings than line graphs (B = 0.22, *p* < 0.001). Directionality and numerical information were not significantly associated. Results are presented in Table 4.

**Fig. 5:**
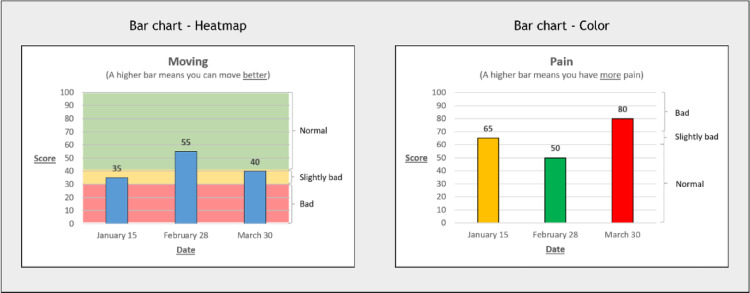
Examples of graphs with high interpretation accuracy and clarity

**Table 3:** Association of different graphical features with interpretation accuracy and clarity

Graphical feature	Interpretation accuracy						Clarity (Q3)			
	Comparison with norm population (Q1)			Changes over time (Q2)						
	OR	*P*-value	95% CI	OR	*P*-value	95% CI	B	SE	*P*-value	95% CI
Numerical information [yes]	0.95	0.528	0.791–1.128	0.82	0.096	0.653–1.035	0.05	0.02	0.68	− 0.044-0.030
Directionality [higher is better]	**1.30**	0.005*	**1.079–1.556**	1.093	0.869	0.869–1.375	0.13	0.02	0.29	0.010–0.084
Format [bar]	**1.23**	< 0.001*	**1.086–1.385**	1.074	0.947	0.947–1.218	**0.22**	**0.01**	< 0.001*	**0.013–0.068**
Concerning score indicators compared to ‘None’										
Heatmap	**2.89^**	< 0.001*	**2.351–3.553**	0.95	0.555	0.789–1.135	**0.48**	**0.02**	< 0.001*	**1.72–0.248**
Color	**1.97**	< 0.001*	**1.655–2.336**	1.01	0.940	0.843–1.203	**0.33**	**0.02**	< 0.001*	**0.107–0.184**
SD-lines	**0.68**	< 0.001*	**0.570–0.804**	**0.65**	< 0.001*	**0.545–0.773**	0.11	0.19	0.098	− 0.236–0.011

Odds ratios of the graphical features are corrected for age of the participant. OR , Odds ratio; Q1 ,  question 1; Q2 , question 2; Q3 , question 3

* and** Bold, **statistically significant result (*p *< 0.05); ^ considered as strong effect (OR < 0.5 or > 2; B > 0.5)

#### Interview single domain graphs

Children often used numerical information, especially when answering the question about changes over time to compare two scores, and in graphs with no concerning score indicators.

Children intuitively felt that graphs should have 0 at the bottom and 100 at the top. Older children could adapt to reversed y-axes and, on further reflection found it logical that lower bars or lines could indicate better outcomes (e.g. for pain). Younger children often kept struggling with reversed y-axes.

Preferences were equally divided between bar graphs and line graphs. Bar graphs were appreciated for their clear and structured visual design, while line graphs were favored for making time comparisons easier.

When concerning score indicators were highlighted by colors, such as in a heatmap, colored bars or colored line dots, children intuitively connected the colors in the graph to the labels on the y-axis and subsequently found it easier to interpret the data. Children often misinterpreted SD-lines and did not use them as cut-offs, which is the way they were intended (e.g., categorizing a score as a ‘little bit bad’ when the score was close to, but below, the SD line).

### Multidomain graphs

#### Interpretation accuracy changes over time

There were no significant differences between graphs with and without numerical information. Percentage correct responses (question two) were highest for multi-domain bar graphs and lowest for radar graphs (Table 5). Post-hoc tests showed that bar graphs had significantly more correct answerers compared to radar graphs (mean difference = 39%, *p* = < 0.001), balloon (mean difference = 23%, *p* = < 0.001), and line graphs (mean difference = 21%, *p* = 0.004). Radar graphs had significantly lower correct response compared to all other graphs.

#### Clarity

Clarity ratings were highest for multi-domain bar graphs and lowest for radar graphs (Table 5).

**Table 5:** Part B descriptives

	Changes over time (Q2), response %			Clarity rating (Q3)
Graph	Correct	Incorrect	*‘I don’t know’*	Mean (SD)
Bar (multidomain)	79	15	6	7.9 (2.2)
Balloon	61	22	18	6.8 (2.6)
Line (Multidomain)	58	24	18	6.8 (2.6)
Radar	41	23	37	5.3 (3.0)

Q2 , question 2; Q3 , question 3

#### Interview multidomain graphs

Bar graphs were preferred as they allowed children to focus on the information of interest, which improved interpretation accuracy. In the balloon graph, they struggled with the use of colors and suggested coloring the grey balloon (previous measurement) for clarity. Multi-domain line graphs were considered overly crowded, making it hard to distinguish individual lines. Children found radar graphs difficult to interpret due to the graph shape and intersecting lines between domains.

### Single versus multidomain graphs

Participants achieved moderately higher accuracy with single-domain line graphs compared to multi domain line graphs (73% vs. 58% correct, effect size *r* = 0.13, *p* < 0.001). Conversely, with bar graphs participants achieved slightly but significantly higher accuracy with multi-domain graphs than with single-domain graphs (79% vs. 74% correct, effect size *r* − 0.05, *p* – 0.02).

#### Interview single versus multidomain graphs

Single-domain graphs that included color-coding were preferred, as they were quick and easy to interpret. The majority of children reported that the multidomain graphs contained too much information, resulting in some children missing information, such as the dates on the y-axis.

### Preferred graph options

Top three most selected graphs for preferred options were (1) bar chart with color (34%), (2) line graph with heatmap (29%), (3) bar chart with heatmap (27%). The least selected option was the radar chart (0.2%).

## Discussion

This is the first study on the effects of PROM visualization on interpretation and clarity in children. Participants approved foundational elements like clear labeling and well-defined axes. In single domain graphs, indicating concerning scores by heatmap or color was associated with higher interpretation accuracy and clarity than no concerning score indicators. Adding SD-lines resulted in lower interpretation accuracy. There were small effects in favor of bar charts. In line graphs, interpretation of directionality ‘higher is better’ was slightly better interpret than ‘higher is more’. For multidomain graphs, bar graphs performed best and radar graphs scored significantly worse than all other graphs. Single-domain graphs with color-coding were most often preferred.

While research in adult patients indicates similar accuracy in interpreting bar and line graphs, most studies recommend using line graphs [12, 13, 16, 18–20]. This may have led to research involving children focusing exclusively on line graphs [21, 22]. However, educational studies indicate that children find bar graphs easier to interpret than line graphs [25, 37, 38] and our results support this, although differences were small. Interview participants were divided on line versus bar graphs, each having perceived advantages and disadvantages. Children are often more familiar with bar graphs as these are introduced earlier in Dutch education than line graphs [23]. Line graphs are seen as more complex as they require an understanding of how a trend is formed by the line rather than discrete categories as in bar graphs [25]. 

Our results on the favorable effects of color use are supported by literature showing that colors help children to memorize patterns or trends and to keep track of changes, thus simplifying interpretation [18, 20, 37]. The poor results of adding SD-lines contrast with previous findings in adults [16]. Our think-aloud participants indicated that children often interpret SD-lines not as cut-off thresholds, but as markers suggesting that scores near the line, rather than beyond it, are of concern. The higher interpretation accuracy of directionality ‘*higher is better’* compared to *‘higher is more’*, corresponds with research in adult patients [16]. However, this result only applies to line graphs, as the y-ax in bar graphs cannot be properly reversed and is therefore not investigated.

Our interview participants recommend using single domain graphs instead of multidomain graphs. Literature shows that children find it difficult to analyze multiple variables at the same time [38]. Although multidomain bar graphs performed similarly to single-domain bar graphs in our data, this result may be attributable to a learning effect. Specifically, questions were randomized only within parts of the test, such that questions from part A (single-domain graphs) were consistently presented before those in part B (multidomain graphs). Other multidomain graphs did not perform as well as bar graphs. Additionally, only single domain graphs were selected as preferred option. Radar graphs were interpreted poorly compared to all other graphs and were rated lowest in clarity and preferred options. We recommend against using this graph type for children because of its complexity.

Although higher correct interpretation rates were found with colors compared to without, application in clinical practice requires caution. Comparisons with normative populations can be confronting for children, especially for those who score below average. During the first year of studying KLIK we experienced that such comparisons could evoke feelings of triumph or disappointment in children [9]. As comparisons to normative data (Q1) are mainly relevant in clinical settings [20], concerning score indicators may be better reserved for consultations. Since these indicators, such as heatmaps, did not improve interpretation accuracy when assessing changes over time (Q2), offering them as an optional feature for home viewing may reduce unnecessary exposure to potentially distressing visuals.

In clinical consultations, PROM visualizations should be used to support dialogue rather than merely to present outcomes [17, 39, 40]. Healthcare professionals can support interpretation by briefly explaining the graph and inviting children to reflect on their own results, for example by identifying current scores or changes over time. When normative comparisons or concerning score indicators are discussed, professionals can provide context and attend to children’s interpretations and emotional responses. Using PROM graphs as a conversation starter may support child-centered communication in pediatric care.

To enhance pediatric patient engagement in health-related discussions in clinical encounters, we aim to implement our findings within the KLIK PROM portal. However, challenges persist. Literature suggests that healthcare professionals prefer line graphs with heatmaps to identify concerning scores [18], whereas our findings indicate a preference for bar graphs among pediatric patients. Ultimately, personalized dashboards that allow patients and professionals to choose their preferred graphical format may best accommodate these differing needs. Together with all stakeholders we have to find a solution to combine all preferences.

This study has limitations. First, we aimed to include a larger sample of pediatric patients for the quantitative study, anticipating that their familiarity with health-related graphical representations would enhance test performance. Although this trend was observed despite the low response rate, the small sample size limits the strength of the evidence. Low response rates were due to technical difficulties during recruitment for the quantitative study, as the test link did not function during a small period of time. Second, participants from the general population were recruited and paid through a recruitment agency, raising the possibility that some children may not have taken the test seriously. However, flatliners were removed to minimize this risk. Third, the number and pattern of data points in the graphs varied across sets, which may have made some graphs easier to interpret than others (e.g., when patient scores were all within the normal range or when the line showed a straight upward trend). This variability may explain differences observed in the raw data. However, in the regression analyses, results from all sets were combined. Fourth, children completed the test at home, which may have allowed for parental assistance, potentially influencing their responses. Finally, this study was conducted in the Netherlands, potentially limiting generalizability. However, while educational curricula may differ across countries, similar progressions in graph literacy are common, supporting the potential transferability of our findings to other settings with comparable educational structures.

## Conclusion

To use PROMs for communication with pediatric patients, it is essential that children can understand their scores. Our results indicate the importance of a well-designed base graph, including foundational elements such as clear labeling and well-defined axes. Bar graphs incorporating heatmaps or color as concerning score indicators are most accurately interpreted by children, and we recommend to use such graphs (as presented in Fig. 5) in clinical encounters with children. As line graphs were interpreted only slightly less accurately than bar graphs, they remain a suitable option for data presentation. For line graphs, the *“higher is better”* directionality is interpreted most effectively by children. We recommend using these features in clinical consultations, where healthcare professionals can provide an explanation and children can be actively involved in discussions about their health.

## Supplementary Information

Below is the link to the electronic supplementary material.

Supplementary Material 1

## Data Availability

Data used for the current study are available upon reasonable request.
