# Supplementary material for: Making sense of PROM outcomes: a mixed method study to optimize graphical visualization formats for children
Source: Qual Life Res. 2026 Feb 12;35(3):66. doi: 10.1007/s11136-026-04172-5 (PMC12901072; doi:10.1007/s11136-026-04172-5)
Supplement: Supplementary file 1 — Supplementary Material 1 [file 11136_2026_4172_MOESM1_ESM.docx]

| **Graphical features** | | | |  |  |  |  |
| --- | --- | --- | --- | --- | --- | --- | --- |
| Concerning_score indicator | Numeric information | Directionality | Format | |  |  |  |
|  |  |  |  |  | Correct % | Incorrect % | I don’t know % |
| Heatmap | Numeric | higher is better | Bar | | 69 | 29 | 2 |
|  |  |  | Line | | 89 | 9 | 2 |
|  |  | higher is more | Bar | | 80 | 19 | 1 |
|  |  |  | Line | | 82 | 16 | 2 |
|  | No numeric | higher is better | Bar* | |  |  |  |
|  |  |  | Line | | 76 | 22 | 2 |
|  |  | higher is more | Bar | | 77 | 22 | 1 |
|  |  |  | Line | | 75 | 24 | 1 |
| Color | Numeric | higher is better | Bar | | 82 | 17 | 1 |
|  |  |  | Line | | 71 | 28 | 1 |
|  |  | higher is more | Bar | | 79 | 20 | 1 |
|  |  |  | Line | | 63 | 33 | 4 |
|  | No numeric | higher is better | Bar* | |  |  |  |
|  |  |  | Line | | 65 | 31 | 4 |
|  |  | higher is more | Bar | | 87 | 13 | 1 |
|  |  |  | Line | | 82 | 17 | 1 |
| SD lines | Numeric | higher is better | Bar* | |  |  |  |
|  |  |  | Line | | 78 | 19 | 3 |
|  |  | higher is more | Bar | | 49 | 47 | 4 |
|  |  |  | Line | | 71 | 26 | 4 |
|  | No numeric | higher is better | Bar | | 69 | 28 | 3 |
|  |  |  | Line | | 47 | 51 | 2 |
|  |  | higher is more | Bar | | 41 | 58 | 1 |
|  |  |  | Line | | 58 | 39 | 3 |
| No concerning scores | Numeric | higher is better | Bar* | |  |  |  |
|  |  |  | Line | | 64 | 31 | 5 |
|  |  | higher is more | Bar | | 81 | 14 | 5 |
|  |  |  | Line | | 50 | 42 | 8 |
|  | No numeric | higher is better | Bar | | 81 | 15 | 4 |
|  |  |  | Line | | 70 | 24 | 6 |
|  |  | higher is more | Bar | | 67 | 29 | 4 |
|  |  |  | Line | | 65 | 28 | 7 |

**Supplementary 1. Overview raw data on correct, incorrect, and *‘I don’t know’* responses of Part A of the test.**

* Raw data is unavailable because bar graphs cannot display reversed y-axes in symptom domains
